# Transcriptomics to study the effect of a Mediterranean-inspired diet on inflammation in Crohn's disease patients

**DOI:** 10.1186/1479-7364-7-24

**Published:** 2013-11-27

**Authors:** Gareth Marlow, Stephanie Ellett, Isobel R Ferguson, Shuotun Zhu, Nishi Karunasinghe, Amalini C Jesuthasan, Dug Yeo Han, Alan G Fraser, Lynnette R Ferguson

**Affiliations:** 1 Discipline of Nutrition, Faculty of Medical and Health Sciences, The University of Auckland Private Bag 92019, Auckland 1142, New Zealand; 2 Nutrigenomics New Zealand, University of Auckland, Private Bag 92019, Auckland 1142, New Zealand; 3 Faculty of Medical and Health Sciences, The University of Auckland, 85 Park Road, Grafton, Auckland 1023, New Zealand; 4 Department of Medicine, University of Auckland, Private Bag 92019, Auckland 1142, New Zealand

**Keywords:** Inflammation, Crohn's disease, Transcriptomics, Dietary intervention

## Abstract

**Background:**

Inflammation is an essential immune response; however, chronic inflammation results in disease including Crohn's disease. Therefore, reducing the inflammation can yield a significant health benefit, and one way to achieve this is through diet. We developed a Mediterranean-inspired anti-inflammatory diet and used this diet in a 6-week intervention in a Crohn's disease population. We examined changes in inflammation and also in the gut microbiota. We compared the results of established biomarkers, C-reactive protein and the micronuclei assay, of inflammation with results from a transcriptomic approach.

**Results:**

Data showed that being on our diet for 6 weeks was able to reduce the established biomarkers of inflammation. However, using transcriptomics, we observed significant changes in gene expression. Although no single gene stood out, the cumulative effect of small changes in many genes combined to have a beneficial effect. Data also showed that our diet resulted in a trend of normalising the microbiota.

**Conclusions:**

This study showed that our Mediterranean-inspired diet appeared to benefit the health of people with Crohn's disease. Our participants showed a trend for reduced markers of inflammation and normalising of the microbiota. The significant changes in gene expression after 6 weeks highlighted the increased sensitivity of using transcriptomics when compared to the established biomarkers and open up a new era of dietary intervention studies.

## Background

Nutrigenomics studies the effect of a specific food or diet on gene and expression using the omic technologies. This will lead to a better understanding of the mechanism of how specific dietary components affect specific gene and proteins resulting in an understanding of how metabolism is influenced [1].

Determining the effects of intervention studies can be difficult, as the change in gene expression for an individual gene may be low but the number of genes affected could be large, resulting in a cumulative beneficial effect as a result of the intervention. Such trials are costly and time consuming and have required a prior hypothesis and ideally pilot data before commencement, to ensure that the correct genes were measured. The technology of transcriptomics has allowed non-hypothesis-based studies to be undertaken. This technique allows the study of thousands of genes simultaneously, and the sensitivity allows the detection of a small change in the expression of a gene. The other advantage of such a sensitive technique is that the results allow intervention trials to be conducted over a much shorter time frame and with fewer participants. The non-hypothesis-based approach and the reduced cost of a shorter smaller trial would allow smaller food companies to prove efficacy of functional foods or novel diets and thus make a substantiated health claim.

Inflammation is a necessary immune response; however, chronic inflammation can lead to chronic diseases including cardiovascular disease, rheumatoid arthritis, Alzheimer's, Crohn's disease and even some cancers [2-4]. Therefore, reducing the inflammation can yield a significant health benefit [2,5,6]. Crohn's disease is a chronic intestinal disorder, typified by chronic inflammation of the gastrointestinal tract. While it has a clear genetic susceptibility, lifestyle factors including diet are important factors in disease development [7-9]. If diet is a cause of symptoms, then it stands to reason that diet can also relieve symptoms, and therefore, Crohn's disease would be a good target for nutrigenomics [1,10].

As Crohn's disease is localised to the gastrointestinal tract, one would expect to find dysbiosis of the intestinal microbiome [11-13]. The interplay between diet, intestinal microbiota, genetics and health is complex [14]. In recent years, the role of the microbiota has become of greater importance in understanding human health and disease. Six bacterial phyla, comprising over 1,000 species, have been identified as the predominant inhabitants of the human gut [15,16]. While previously considered a static entity, it is now emerging that the ratio of the bacterial phyla and species in the gut is constantly changing in response to external influences like diet. Different diseases also have a deviation from what is being considered a 'normal’ ratio of bacterial phyla [17]. Whether change to the microbiota is a cause or effect of disease is currently unknown.

A healthy intestinal mirobiome consists of six bacterial phyla—Firmicutes, Bacteroidetes, Actinobacteria, Proteobacteria, Fusobacteria and Verrucomicrobia, with species of Firmicutes and Bacteriodetes being the most abundant at approximately 65% and 25%, respectively. Variations are observed in healthy individuals by the abundance of the different species within each phylum; however, disease association is observed by variations in the abundance of the six phyla [18]. Manichanh [19] and Sokol [20] both reported a reduction in faecal microbial diversity of patients with Crohn's disease, particularly a reduction in the number of species of the phyla Firmicutes (specifically *Clostridium* clusters IV and XIVa) and Bacteroidetes, with a corresponding increase in Proteobacteria and *Bacillus*[21] (Table 1). We developed an anti-inflammatory diet based upon a Mediterranean-inspired diet, which is well-known for its protective ability towards chronic disease [2,5,22-26]. To this base diet, we added foods that previous research has shown to be beneficial in reducing inflammation and removed foods that are known to be detrimental to Crohn's disease patients [27,28]. We conducted a pilot study in early 2012 [29], using both the established validated biomarkers, C-reactive protein (CRP) [30,31] and micronucleus assay (MN) [32], and transcriptomics to test the ability of this Mediterranean-inspired diet to reduce inflammation in healthy people [29]. Based on the success of this trial as determined by a statistically significant reduction in CRP in this healthy group, a similar trial was undertaken in participants with Crohn's disease. In this second study, we hoped to prove that the diet reduced inflammation and was beneficial to patients with Crohn's disease. We also hoped to show that the transcriptomic technique was more sensitive and thus required fewer subjects over a shorter time frame than the established biomarkers [5,33,34].

**Table 1:** The relative abundance of bacteria in a healthy and Crohn's disease microbiome

**Bacterial phyla**	**'Healthy’ microbiome (%)**	**'Crohn's disease’ microbiome**
Bacteroidetes	Approximately 25	Decreased expression
Firmicutes	Approximately 65	Decreased expression in *Clostridium* clusters IV and XIVa and increased expression in *Bacillus*
Actinobacteria	Approximately 5	
Proteobacteria	Approximately 8	Increased expression
Fusobacteria	Approximately 1	
Verrucomicrobia	Approximately 2	

## Results

### Adherence to the diet

All eight participants completed the 6-week diet and in a self-reported questionnaire felt they adhered to the diet well. Based on participant responses to the food diaries, we were able to show that the average total energy consumption reduced from 8,189 to 6,716 kJ, and cholesterol levels were reduced by 20%. There was also a reduction in average intake of saturated fat (43.9% to 32.1%) and a corresponding increase in mono- (38.9% to 46.6%) and poly-unsaturated fats (17% to 21.1%).

### Biomarkers

**Figure 1:**
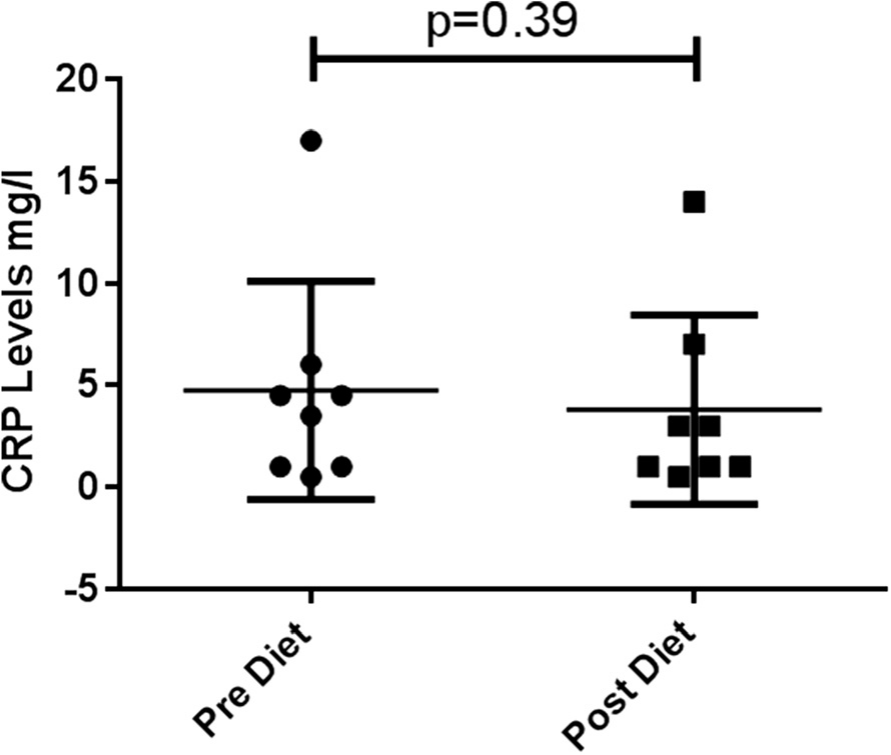
Mean (±SE) levels of C-reactive protein pre- and post-dietary intervention for each participant.

**Figure 2:**
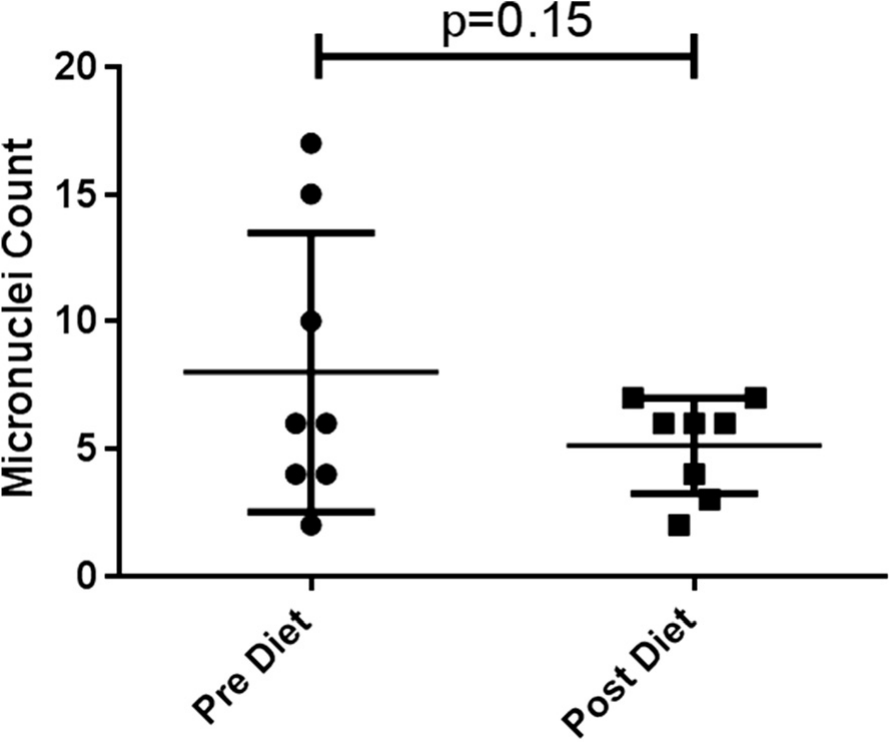
Mean (±SE) micronuclei scored in 1,100 cytokinesis-blocked cells for each participant pre- and post-dietary intervention.

The established biomarkers, CRP and micronuclei numbers, showed a trend of reduction after the 6-week diet; however, this was not significant. The change in CRP level as a result of dietary intervention is shown in Figure 1. Overall, there was a reduction after the 6-week diet, but this was not statistically significant (*p* = 0.39). Cytokinesis-blocked micronuclei numbers before and after dietary intervention are shown in Figure 2. Although numbers decreased after intervention, this reduction did not reach statistical significance (*p* = 0.15).

### Gene expression

**Figure 3:**
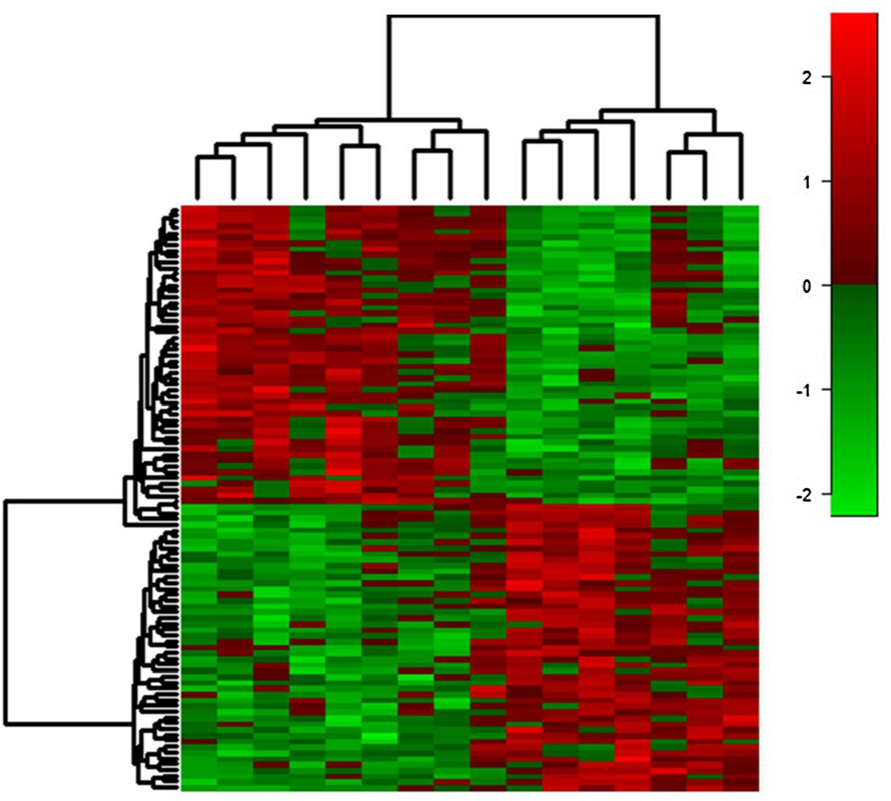
**Differential expression of top 100 genes (*p*** **< 0.002) pre- and post-dietary intervention.** Based on analysis of the top differentially expressed transcripts, it is clear that transcription expression is changed over time, with expression being both increased (*red*) and decreased (*green*) significantly in just 6 weeks.

Using the Affymetrix PrimeView™ microarray (Santa Clara, CA, USA), we found that gene expression was significantly affected (Figure 3). In total, we found that 3,551 genes had significantly (*p* < 0.05) altered expression as a result of the dietary intervention, with 1,902 genes being up-regulated and 1,649 down-regulated. These results highlight the increased sensitivity in this methodology.

Just changing gene expression is of no benefit; we expect our diet to change inflammation-relevant genes. We used pathway analysis software (Ingenuity Pathway Analysis (IPA)) to examine these differentially expressed genes in more detail. Table 2 shows the top functions and the number of differentially expressed genes for each. The functions were grouped into three categories by IPA: (1) diseases and disorders, (2) molecular and cellular function and (3) physiological system development and function.

**Table 2:** Key functions associated with dietary intervention

**Top function**	**Number of genes**	***p *** **value**
Diseases and disorders		
Neurological disease	122	8.67E - 09 to 9.70E - 03
Skeletal and muscular disorders	138	8.67E - 09 to 7.60E - 03
Infectious disease	126	8.67E - 08 to 1.41E - 02
Psychological disorders	91	1.27E - 05 to 9.70E - 03
Cancer	358	1.37E - 05 to 1.37E - 02
Molecular and cellular function		
Cellular growth and proliferation	218	1.23E - 07 to 1.47E - 02
Cellular development	177	1.81E - 07 to 1.40E - 02
Cell cycle	84	9.24E - 07 to 1.24E - 02
Cell-to-cell signalling and interaction	108	1.76E - 06 to 1.46E - 02
Cellular movement	120	1.76E - 06 to 1.37E - 02
Physiological system development and function		
Hematological system development and function	136	1.76E - 06 to 1.40E - 02
Immune cell trafficking	66	1.76E - 06 to 1.24E - 02
Tissue development	124	1.76E - 06 to 1.46E - 02
Organismal survival	154	3.81E - 06 to 3.81E - 06
Cardiovascular system development and function	110	3.90E - 06 to 1.32E - 02

Table 3 summarises the significantly affected canonical pathways for the dietary intervention, calculated by IPA. The results are ranked according to significance (Fischer's exact test). The ratio for each pathway is also given. The ratio is the number of genes in the dataset that are in the canonical pathway divided by the total number of genes in that pathway.

**Table 3:** Key canonical pathways affected by intervention

**Name**	***p *** **value**	**Ratio**
EIF2 signalling	4.24E - 04	17/200 (0.085)
B cell development	9.31E - 04	6/33 (0.182)
T helper cell differentiation	3.55E - 03	8/72 (0.111)
Uracil degradation II (reductive)	7.6E - 03	2/11 (0.182)
Thymine degradation	7.6E - 03	2/11 (0.182)

**Figure 4:**
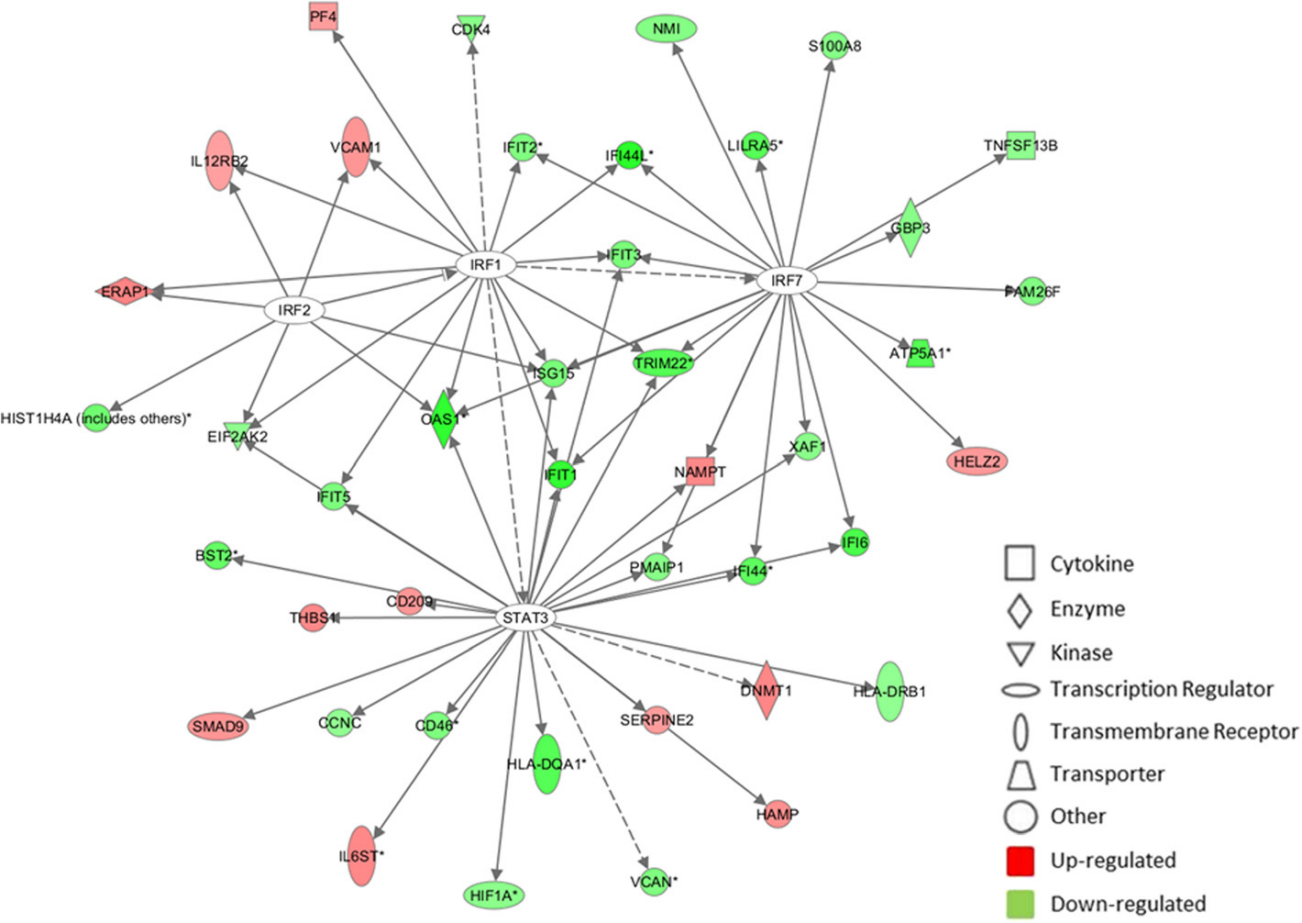
**Generation of a biological network of genes related to the upstream regulator IRF2.** Network was generated by IPA. Connections were applied based on known interactions within the Ingenuity Pathway Knowledge Base. *Solid lines* between genes represent direct interactions and the *dashed lines* indirect. Genes are represented by *nodes*, with the *red* and *green colours* indicating up- or down-regulated expression; the greater the colour intensity, the higher the level of differential expression.

In order to better understand the connections between the differentially regulated genes, we mapped the genes into a network diagram based on interactions as determined by the IPA Knowledge Base, using the upstream regulator interferon regulatory factor 2 (IRF2) (Figure 4). IRF2 has been shown to regulate NF-κB activity, which is essential in control of immune response and inflammation [35] and within the network to indirectly interact with signal transducer and activator of transcription 3 (STAT3). STAT3 is an important transcription factor in the JAK/STAT pathway and has been shown to be affected in inflammatory bowel disease (IBD) [36,37].

### Microbiota abundance

**Figure 5:**
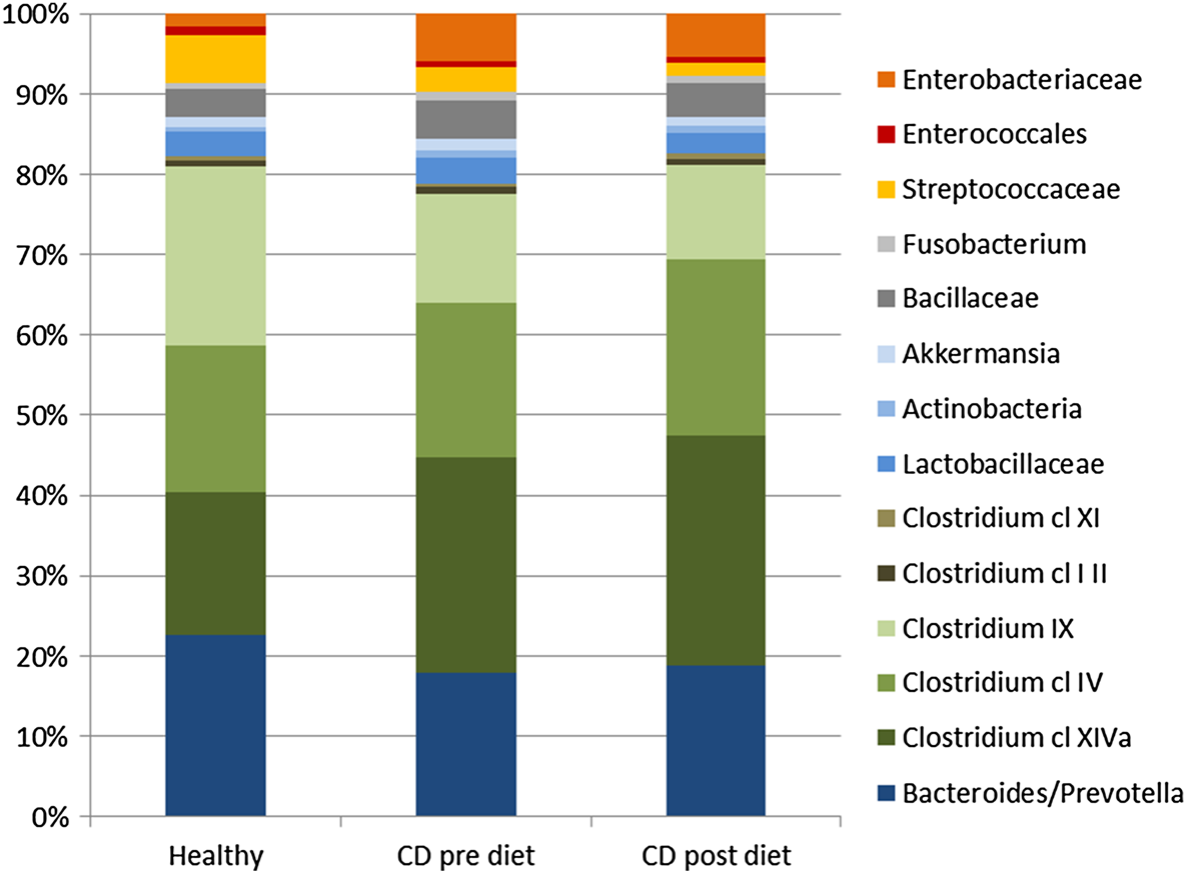
**Relative abundance of microbiota pre- and post-dietary intervention.** Healthy samples (*n* = 2), CD pre-diet (*n* = 8) and CD post-diet (*n* = 8).

The expression of the six bacterial phyla of the study participants was not too dissimilar from the healthy specimens (Figure 5) or from the theoretical diversity of a normal microbiome. However, before the diet, there was a tendency towards a reduction in bacterial diversity, indicative of Crohn's disease. This is noted by a combined relative abundance of Bacteroidetes at 17.89% in the study participants compared to 22.64% in the healthy individuals, Bacillaceae at 4.65% compared to 3.44% in the healthy individuals and a 5.93% relative abundance of Proteobacteria in the study participants compared to 1.67% in the healthy individuals. The relative abundance of Firmicutes was unchanged between the study group (72.8%) and the healthy individuals (73%).

When combining all data from Crohn's disease participants (*n* = 8) and making pre- and post-diet comparisons of each bacterial cluster, there is a forward trend, yet no significant improvement in the expression of the bacteria that were known to have an altered relative abundance in Crohn's disease (Figure 5). There was an increase in the expression of Bacteroidetes (17.89% to 18.74%), *Clostridium* cluster IV (19.2% to 21.86%) and *Clostridium* cluster XIVa (26.78% to 28.79%) and a decrease in the abundance of Proteobacteria (5.93% to 5.48%) and Bacillaceae (4.65% to 4.21%).

## Discussion

As previously stated, inflammation can cause chronic disease; therefore, it is important to identify and reduce inflammation, one such way is through diet. We found that in this pilot study, there was a small reduction in the established biomarkers of inflammation after consuming our diet for 6 weeks. Using the transcriptomic approach, we did see significant changes in gene expression. In total, we found 3,551 genes that were differentially expressed in our study as a result of a 6-week dietary intervention. The most common candidates were not the genes that were affected, and although results for individual genes were not spectacular, it is the combination of affected genes that is interesting. The data highlighted the difference in sensitivity between the established biomarkers and the transcriptomic approach and confirmed that conducting a short-term trial with a small number of participants is feasible.

The patients who volunteered for this study were selected based on strict criteria. Several of them already used diet as a way of controlling symptoms and so for transcriptomics to still show an effect highlights how sensitive this technique is. This increased sensitivity allows for short-term studies with fewer study numbers than traditional trials to be performed, which has significant implications for future human clinical trials.

The ratio of the six bacterial phyla or families from the faecal microbiome of healthy individuals has been determined. It is well documented that deviation from this 'norm’ occurs in individuals who have Crohn's disease. Normalising the intestinal microbiota through diet is a step towards potentially reducing inflammation and thus restoring health and improving the quality of life for people with Crohn's disease.

After 6 weeks on our Mediterranean-inspired diet, there was some 'normalising’ of the intestinal microbiota. As previously stated, the participants were already managing their disease through diet, so the expression of the microbiota was not too dissimilar from that documented in a healthy group. However, as a group, the microbiota before the diet was tending towards that expected for people with Crohn's disease, notably a reduction in the presence of Bacteroidetes and an increase in the presence of Proteobacteria and Bacillaceae.

Consuming the Mediterranean-inspired anti-inflammatory diet for 6 weeks was able to influence the intestinal microbiota of our study population, although this improvement should be noted as a forward trend rather than any statistically significant change. There was an observed increase in Bacteroidetes and the *Clostridium* clusters and a decrease in Protebacteria and Bacillaceae.

## Conclusions

One of the aims of this pilot study was to compare the data obtained from traditional biomarkers of inflammation with that of transcriptomics. The Affymetrix GeneChip® PrimeView™ arrays were selected as they provide complete coverage of the human genome. We were impressed by the increased sensitivity provided by the arrays over the biomarkers.

Peripheral blood mononuclear cells (PBMCs) can be easily obtained from a venepuncture, which allows for easy and repeated collection unlike biopsies of other tissues [38]. PBMCs have been used to examine gene expression in numerous diseases including inflammation [39-45] and have also been shown to act as a biomarker of nutritional interventions [22,46,47] and used to predict inflammatory response to functional foods [48]. The increased sensitivity of transcriptomics and the fact that PBMCs can be used as a model to study the expression of inflammatory genes [40] make this methodology attractive for non-hypothesis-based studies.

One of the drawbacks of this study was the small study population, which resulted in observing a trend in effect of the diet rather than significant results. However, one of the benefits of using transcriptomics is the ability to conduct dietary interventions over a shorter time frame and requiring fewer subject numbers. This subsequently reduce costs which will open up the area of health-based food claims to a wider market, meaning that more companies and more products could be tested. This could lead to a better understanding of the mechanism of action and thus substantiated health claims benefiting both the companies and the population.

The data from this pilot study, although not significant, does highlight that by choosing an anti-inflammatory diet, even for a short time, can have an effect on reducing inflammatory markers, changing gene expression and normalising the microbiota.

## Methods

### Study design

This study was granted ethical approval by the Northern Regional Ethics Committee of New Zealand (reference NTY/11/11/109). Requirements for inclusion in the study were very strict and were intentionally so given the aetiology of Crohn's disease and the potential bias from medication and surgical interventions. Selection criteria included the following: had no bowel surgery, was not taking prednisone or similar anti-inflammatory medication, had no changes to their medication over the last 3 months, indicating a stable condition, were willing to provide samples at the beginning and end of the study and were willing to change their diet. This was self-reported by the participants.

All participants consumed the Mediterranean-inspired anti-inflammatory diet for 6 weeks. They were provided with food items including salmon, organic avocados, kumara, a variety of vegetables, gluten-free bread, New Zealand extra virgin olive oil, green tea, honey and fish oil capsules. The majority of these foods were grown and produced in New Zealand and were used to supplement the participant's diet. A reduction in the consumption of red meat, white flour, high-fat foods and sugar was also expected. All participants provided a blood and faecal sample at the beginning and end of the study and completed food diaries.

### Subject selection/eligibility

Eight participants, from Auckland, New Zealand, who had active yet stable Crohn's disease symptoms and passed the selection criteria (see above) were selected to participate following an initial invitation to all Crohn's disease participants in the Diet and Gene Database administered by Nutrigenomics New Zealand.

Our participants were six females and two males, aged between 31 and 60 years (mean age 45.4 years), and the length of time since diagnosis with Crohn's disease ranged from 7 to 36 years.

### Study outcomes

Levels of C-reactive protein, a systemic measure of inflammation [31] and changes in DNA damage in peripheral blood lymphocyte cells by the micronucleus assay (in accordance with OECD guidelines 487) were scored before and after the diet, along with comparisons of RNA gene expression profiles.

### C-reactive protein

A blood sample collected in serum-separating tubes was analysed for C-reactive protein. C-reactive protein is found in the blood in response to inflammation and is a biomarker for potential disease development. Specimens were analysed by a medical testing laboratory, Labtests Auckland, according to their protocols for the latex-enhanced immunoturbidimetric assay.

### DNA damage—micronucleus assay

The cytokinesis-block micronucleus assay [32], a validated method for determining DNA damage in peripheral blood lymphocytes cells, was used to observe any changes in DNA damage before and after the diet intervention. DNA damage was determined by the presence of micronuclei (MN) in the cytoplasm of bi-nucleated lymphocyte cells. Whole blood treated with anticoagulant (heparin) was collected, stored at room temperature and cultured within 24 h. Duplicate cultures containing 0.6 ml whole blood and 9.25 ml alpha MEM and 15% foetal calf serum (Gibco New Zealand Ltd., Auckland, New Zealand) were prepared, to which 150 μl phytohaemaglutinin (PHA, Gibco New Zealand Ltd.) was added to stimulate lymphocyte replication. The cells were incubated in a humidified incubator containing 5% carbon dioxide for 44 h. Cytochalasin B (Sigma Chemical Co., St. Louis, MO, USA) was added to give a final concentration of 4.5 μg/ml, and the culture was incubated for an additional 26 h. The cytochalasin B would halt any further cell growth. Cells were harvested using standard protocols as outlined [49], including centrifugation, swelling of the cells using hypotonic KCl and fixing in a solution of methanol and acetic acid before dropping onto slides. Once dried, the slides were stained with Diff-Quik (Dade Behring Inc., Sacramento, CA, USA) according to staining protocols. Two thousand bi-nucleated cells were scored for micronuclei using the criteria outlined by Heddle et al. [50] and Fenech [51].

### Transcriptomics

#### RNA isolation and storage

Whole blood was collected in a PAXgene Blood RNA Tube (PBRT) (PreAnalytiX) following the manufacturer's recommended protocol. The blood was incubated at room temperature for 2 h, after collection to improve RNA yield, before being stored at -70°C.

RNA extraction was performed according to the recommended protocol PAXgene Blood RNA System Kit (Qiagen). The quantity of RNA was determined using the NanoDrop ND-1000 Spectrophotometer (NanoDrop Technologies, Wilmington, DE, USA) and the quality of RNA by the Experion RNA StdSens Analysis Kit (Biorad, Hercules, CA, USA). For successful gene expression microarray hybridization, a concentration of >100 ng/μl of RNA and a RNA quality indicator (RQI) of >8 are required.

#### Gene expression arrays

Microarray hybridization was performed on GeneChip® PrimeView™ Human Gene Expression arrays (Affymetrix) following the manufacturer's protocol by The Ramaciotti Centre, University of New South Wales, Sydney, Australia, using the Affymetrix GeneChip® Instrument System.

### Gene expression data analysis

Affymetrix Primeview array data was read using the 'affy’ package in the statistical language R and normalised using the RMA method. QA of the data showed that it was of good quality and free of obvious artifacts or outliers. The 'limma’ package was used to compare gene expression pre- and post-diet, paired by participant code.

Further analyses of the differentially expressed genes, including network, pathway and functional analysis, were carried out using IPA (Ingenuity Systems Inc., USA; http://www.ingenuity.com).

### Microbiota analysis

A faecal sample was provided by each study participant before and after the diet. The samples were frozen at -80°C until required for analysis. Total DNA from the faecal matter was extracted according to the protocol published by Candela et al. [18] in combination with the DNAeasy Blood and Tissue Kit 50 (Qiagen). The final DNA concentration and purity were determined using the NanoDrop ND-1000 Spectrophotometer (NanoDrop Technologies). The extracted DNA was standardised to 50 ng/μl and stored at -20°C. These samples were then sent to the Department of Pharmaceutical Science, University of Bologna, Italy for analysis. The amplification and purification of DNA and the subsequent HTF-Microbi.Array were conducted according to protocols defined by Candela et al. [18]. Briefly, amplification of the DNA by PCR was achieved using 16 s rDNA sequencing, facilitated by Taq polymerase (Promega, Madison, WI, USA) and the universal forward primer f27 (5′-AGAGTTTGATCMTGGCTCAG-3′) and reverse primer 1492r (5′-TACGGYTACCTTGTTACGACTT-3′). The resultant PCR product was purified using the PCR Clean-up System Kit (Promega) and concentration of the purified DNA determined by nanodrop.

Specific detection and quantification of 32 phylogenetically related groups of the human intestinal microbiome were achieved by the HTF-Microbi.Array, an adaption to the Ligase Detection Reaction [18]. DNA probes specific for each of the currently identified bacteria of the intestinal microbiome are embedded on the array slide. Successful hybridisation of the DNA product to the probe results in fluorescence. The fluorescence intensities (IF) were normalised to the ligation control signal and background IF values, removing outlier signals that were 2.5-fold above or below the average of the repeated signals (four repeats of each probe on every array). The results were compared to two healthy control samples which were obtained at the start of the study.

## Abbreviations

CRP: C-reactive protein; IBD: Inflammatory bowel disease; IPA: Ingenuity pathway analysis; IRF2: Interferon regulatory factor 2; JAK: Janus kinase; KCl: Potassium chloride; MEM: Minimum essential media; MN: Micronuclei; NF-κB: Nuclear factor kappa-light-chain-enhancer of activated B cells; PBMCs: Peripheral blood mononuclear cells; PBRT: PAXgene Blood RNA Tube; RMA: Reliabilty, maintainability and availability; RQI: RNA quality indicator; STAT3: Signal transducer and activator of transcription 3.

## Competing interests

The authors declare that they have no competing interests.

## Authors' contributions

GM and ACJ carried out the gene expression studies. SE carried out the microbiota analysis. SZ, NK and IRF carried out the biomarker work. DYH participated in the design of the study and performed the statistical analysis. GM and SE participated in study design and drafted the manuscript. LRF and AGF conceived of the study and participated in its design and coordination and helped draft the manuscript. All authors read and approved the final manuscript.
